# Attenuating trabecular morphology associated with low magnesium diet evaluated using micro computed tomography

**DOI:** 10.1371/journal.pone.0174806

**Published:** 2017-04-03

**Authors:** Shu-Ju Tu, Shun-Ping Wang, Fu-Chou Cheng, Chia-En Weng, Wei-Tzu Huang, Wei-Jeng Chang, Ying-Ju Chen

**Affiliations:** 1 Department of Medical Imaging and Radiological Sciences, College of Medicine, Chang Gung University, Tao-Yuan, Taiwan; 2 Department of Medical Imaging and Intervention, Linkuo Chang Gung Memorial Hospital, Tao-Yuan, Taiwan; 3 Department of Orthopedics, Taichung Veterans General Hospital, Taichung, Taiwan; 4 Department of Life Science, Tunghai University, Taichung, Taiwan; 5 Stem Cell Center, Department of Medical Research, Taichung Veterans General Hospital, Taichung, Taiwan; 6 National Laboratory Animal Center, National Applied Research Laboratories, Taipei, Taiwan; 7 Department of Food and Nutrition, Providence University, Taichung, Taiwan; University of Campinas, BRAZIL

## Abstract

**Objective:**

The literature shows that bone mineral density (BMD) and the geometric architecture of trabecular bone in the femur may be affected by inadequate dietary intake of Mg. In this study, we used microcomputed tomography (micro-CT) to characterize and quantify the impact of a low-Mg diet on femoral trabecular bones in mice.

**Materials and methods:**

Four-week-old C57BL/6J male mice were randomly assigned to 2 groups and supplied either a normal or low-Mg diet for 8weeks. Samples of plasma and urine were collected for biochemical analysis, and femur tissues were removed for micro-CT imaging. In addition to considering standard parameters, we regarded trabecular bone as a cylindrical rod and used computational algorithms for a technical assessment of the morphological characteristics of the bones. BMD (mg-HA/cm^3^) was obtained using a standard phantom.

**Results:**

We observed a decline in the total tissue volume, bone volume, percent bone volume, fractal dimension, number of trabecular segments, number of connecting nodes, bone mineral content (mg-HA), and BMD, as well as an increase in the structural model index and surface-area-to-volume ratio in low-Mg mice. Subsequently, we examined the distributions of the trabecular segment length and radius, and a series of specific local maximums were identified. The biochemical analysis revealed a 43% (96%) decrease in Mg and a 40% (71%) decrease in Ca in plasma (urine excretion).

**Conclusions:**

This technical assessment performed using micro-CT revealed a lower population of femoral trabecular bones and a decrease in BMD at the distal metaphysis in the low-Mg mice. Examining the distributions of the length and radius of trabecular segments showed that the average length and radius of the trabecular segments in low-Mg mice are similar to those in normal mice.

## Introduction

Bones are critical organs for physically supporting and protecting internal organs in humans. Muscles and bones are interconnected as the musculoskeletal system, which is essential for daily biomechanical function. In addition to functioning as the reservoir of minerals, bones are responsible for the production of red and white blood cells. Previous studies involving humans and small animals have shown that the optimal combination of nutrients and mineral supplements is essential for effective bone development [1–6]. In particular, insufficient dietary intake of specific minerals, such as Mg, can have physical and biological consequences on the microstructure, bone mineral density (BMD), and bone mineral content (BMC) in trabecular bones.

Trabecular bone is also called cancellous or sponge bone. In humans, trabecular bones and cortical bones respectively account for approximately 20% and 80% of all bone cells [5,7]. Typically, trabecular bones are located at the ends of the femur or tibia and the interior of the vertebrae. Compared with cortical bones, trabecular bones are less dense, more flexible, and possess a more complex geometrical structure. Biomechanically, the function of trabecular bones is associated with resistance to compressive, tensile, and shear-force impacts. Biologically, trabecular bones are metabolically more active in exchanging Ca ions and are remodeled more rapidly during physiological processes than cortical bones [5].

Previous studies investigating rodent models with severely deficient Mg diets (0.04% of nutrient requirements) have observed impaired bone growth, increased loss of bone mass, and an increased risk of skeletal fracture [1–3]. A study involving a rat model with temperate low-Mg diets (10% of nutrient requirements) observed a decrease in bone volume and trabecular number by using a histomorphometric technique [8]. Another study investigating the dietary effect of temperately and severely low Mg intake on bone composition and metabolism in young growing rats reported aberrant bone turnover and a reduction in Mg concentration [4]. A nationwide study conducted in Norway reported a correlation between the concentration of Mg in drinking water and the incidence of hip fractures in humans [6].

In clinical applications, dual-energy X-ray absorptiometry (DXA) has been the standard imaging modality for examining areal BMD and BMC [9,10]. However, DXA images are restricted to 2-dimensional X-ray radiography, and no 3-dimensional information on the microstructure of trabecular bones is available [9,10]. In recent years, microcomputed tomography (micro-CT) technology has advanced because of developments in high-speed image reconstruction. The spatial resolution of micro-CT images has improved to the scale of a few microns [11–13]. In particular, images acquired through micro-CT are isotropic and truly 3-dimensional, in contrast to the 2-dimensional images obtained through X-ray radiography or DXA [11–13]. The main purpose of our study is the 3-dimensional imaging approach of micro CT to quantitatively evaluate the morphological phenotype of femur trabeuclars in mice with a low magnesium diet. In particular, we developed new quantitative measurements of the statistical distribution for trabecular segment length and radius. These statistical distributions allow us for further accurate assessment of trabecular bone development. The biochemical analysis of Mg and Ca in plasma and urine was included to support the results of micro CT assessment. The imaging approach of DXA was not included, mainly the result of DXA is limited to an image space of 2-dimensional projection and quantitative assessment of trabecular morphology with high precision is not suitable.

In this study, we used a micro-CT scanner (SkyScan 1076, Bruker micro CT, Belgium) to investigate the physical characteristics of bone development in femoral trabecular bones between C57BL/6J mice with a basal diet and those with a low-Mg diet over an 8-week period. Technical assessment of the micro-CT images included calculating the BMD, BMC, and quantitative parameters derived from the microarchitecture of trabecular bones [14]. A standard phantom was employed for calibrating and calculating the corresponding BMD (measured in mg-HA/cm^3^) and BMC (measured in mg-HA). In addition to the imaging evaluation of the trabecular bones, a biochemical analysis of Mg, Ca, and P levels in plasma and urinary excretion was conducted for a comparative examination.

## Materials and methods

Four-week-old C57BL/6J male mice were purchased from BioLASCO Taiwan Co., Ltd. (Taipei, Taiwan) and maintained in a temperature and light-controlled room (12 h light, 12h of darkness). Animal care and experimental procedures were approved by the Institutional Animal Care and Use Committee of Taichung Veterans General Hospital (ID: La-1021102). All mice were supplied a basal diet over a one-week acclimatization period. Subsequently, the mice were randomly assigned to 2 groups, each group containing 8 mice, and maintain on one of the following two diets: the basal diet and a low-Mg diet. The control group was supplied the basal diet (5755 TestDiet containing 0.7 mg/g Mg, Richmond, IN, USA), and the low-Mg mice were supplied a low-Mg diet (5865 TestDiet containing <0.08 mg/g Mg) for 8 weeks. Distilled water and food were available ad libitum. On the final day of the experiment, the mice were placed individually into stainless-steel metabolic cages. Urine was collected from each mouse for 24 h to determine the Mg, Ca, and P concentrations. Subsequently, the mice were sacrificed and blood samples were collected for a biochemical analysis of the Mg, Ca, and P levels in plasma. Finally, the femur bones were removed for a quantitative micro-CT assessment.

The imaging system used in this study was a desktop micro-CT scanner (SkyScan 1076, Bruker micro CT, Belgium), in which the X-ray tube and detector were housed and integrated in a radiation-shield instrument. The X-ray beam was collimated as a cone-beam irradiation system. The detector was a charge-coupled device camera with a resolution of 11 million pixels. The energy capacity of the electric potential for accelerating the electrons to bombard the anode of metal target was between 20 and 100 kV. A modified high-speed Feldkamp algorithm in the software was used for image reconstruction [15,16].The animal bedding was composed of a carbon fiber material. In this study, the micro-CT scanner was operated at 50 kV, and a 0.5-mm-thick Al filter was used to obtain the optimal image contrast. Using circular scanning, 360 project images were acquired with a total of 360 rotational steps. Prior to the image analysis and quantitative calculations, the images were reconstructed and processed at a spatial resolution of 9.0 μm. Coefficients of variation were used to represent the variability for each morphological parameter.

**Fig 1:**
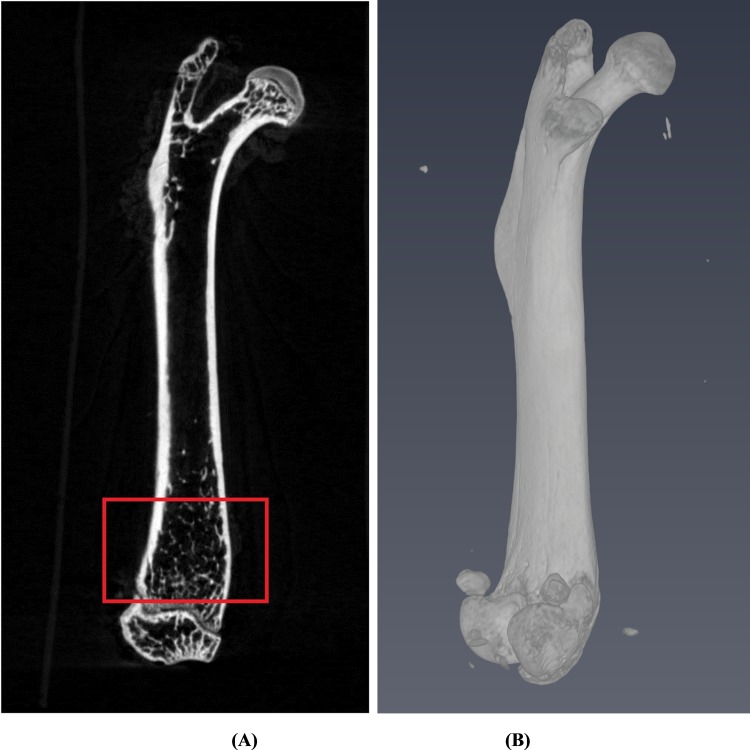
(A) The growth plate was first located at the distal femur site as a reference plane and delineated a 2 mm distance along the longitudinal direction as shown in the red box for our region of interest. (B) A 3-dimensional surface rendering figure of the whole femur bone which is reconstructed from micro CT images is shown.

We used the growth plate in the distal femur bone as a reference because it is an easily identifiable anatomical site [17,18]. For the image analysis, a region of interest (ROI) was selected and delineated into 2-mm segments along the longitudinal direction. The vertical distance between the first image in the ROI and the growth plate was 0.5 mm. The region of interest in our quantitative image analysis and the corresponding 3D image with computer surface rendering are shown in Fig 1.

CTAn (CT-Analyzer, Bruker micro CT, Belgium), Avizo (Visualization Sciences Group, Massachusetts, USA), and ImageJ (National Institute of Health, Maryland, USA) were employed to conduct the ROI delineation and quantitative image analysis. The assessment of architecture-related parameters involved evaluating the total trabecular length, total tissue volume (mm^3^), bone volume (mm^3^), percent bone volume, fractal dimension, structural model index, number of trabecular segments, trabecular segment density (mm^-3^), mean trabecular segment length (mm), mean trabecular segment radius (mm), number of connecting nodes, connecting node density (mm^-3^), and surface-area-to-volume ratio [18]. We also examined the distributions of the length and radius of the trabecular segments. A quantitative analysis was performed using computational algorithms for Euclidean distance mapping and ray tracing [19–21]. In this study, trabecular bones were regarded as an intricate structure interconnected by trabecular segments. A trabecular segment was mathematically modeled as a cylindrical rod, and a connecting node was considered the location where trabecular segments connect. Table 1 lists a summary of the quantitative parameters.

**Table 1:** Quantitative parameters for the imaging analysis of femoral trabecular bones.

Parameters	Standard Unit	Definition and Description	Low-Mg vs Basal
Total tissue volume	mm^3^	Measurement of entire volume of region-of-interest in distal-metaphysis with a vertical length of 2.0 mm. The separation between the growth plate and total tissue volume is 0.5 mm.	Decrease
Bone volume	mm^3^	Volume measurement of trabecular bones inside total tissue volume.	Decrease
Percent bone volume	%	Percent ratio of trabecular bone volume inside total tissue volume.	Decrease
Fractal dimension		Geometric complexity in the non-integer dimensionality. The fractal dimension in our analysis represents how trabecular bones occupy the space.	Decrease
Structural model index		Relative prevalence between a rod and plate. The index is between 0 and 3. 0 represents a 2D plate and 3 for a 3D cylindrical rod.	Increase
Trabecular segment number		Total trabecular segment number in the total tissue volume. The trabecular bone is modeled as a cylindrical rod.	Decrease
Trabecular segment number density	mm^-3^	Total number of trabecular segment divided by the measurement of total tissue volume	Decrease
Mean trabecular segment radius	mm	The average radius for total trabecular segments	Similar
Mean trabecular segment length	mm	The average length for total trabecular segments	Similar
Connecting node number		Total number of the branch node which is inter-connected by different trabecular segments.	Decrease
Connecting node number density	mm^-3^	Total number of connecting node divided by the total tissue volume	Decrease
Surface area to volume ratio	mm^-1^	Amount of surface area per unit volume for trabecular bones	Increase
Bone mineral density	mg-HA/cm^3^	Measurement of bone mineral content in mg-HA in total tissue volume divided by the total tissue volume	Decrease
Bone mineral content	mg-HA	Total bone mineral content in mg-HA in the total tissue volume	Decrease

A standard bone phantom (QRM-microCT-HA, QRM GmbH, Moehrendorf, Germany) for BMD measurement was calibrated and applied to calculate the BMD of the femoral trabecular bones [22,23]. The phantom comprised 5 cylindrical inserts of known densities of Ca hydroxyapatite, Ca_10_(PO_4_)_6_(OH)_2_. A proprietary epoxy resin was uniformly filled as the base material. The 5 BMD values for each insert were 200, 400, 600, 800, and 1000 mg-HA/cm^3^. The cylinders were 32 mm in diameter and 38 mm in length, whereas each insert was 5 mm in diameter and 38 mm in length.

Mg, Ca, and P levels in plasma were determined using an automated clinical chemistry analyzer (TBA-200 FR, Toshiba Medical Products, Tokyo, Japan), and those in urine samples were measured using a biochemistry automatic analyzer (HITACHI 7180, Hitachi Medical Corporation, Tokyo, Japan). Calculations are presented as the mean ± standard error of the mean (SEM). A *t* test was performed for statistical analysis, and a *z* test was used to determine whether the equal-variance assumption should be applied. Results were considered statistically significant where *P*<0.05.

## Results

### 3-dimensional visualization of a femur bone from micro CT images

**Fig 2:**
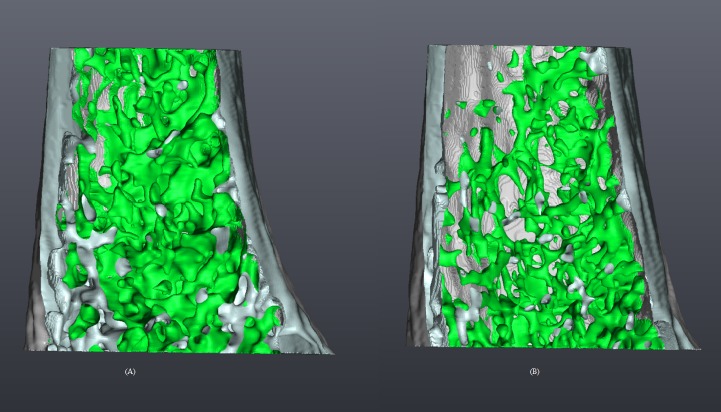
**Volumetric micro-CT imags of a femoral bone, depicting the trabecularbone (green) and cortical bone (gray) of a mouse in the control (A) and low-magnesium (B) group.** Surface renderingwas employed to process the images.

Fig 2A and 2B show micro-CT images (spatial resolution, 9 μm) representing typical trabecular bones of the mice in the control and low-Mg diet groups, respectively. Surface rendering was employed to produce the volumetric images [24,25]. The figures reveal a lower population of trabecular bones at the distal metaphysis in the low-Mg mice.

### Measurement of vertical length for trabecular bones

To estimate a quantity for the global structure of trabecular bones at the distal metaphysis, we defined a geometric measure for vertical length. A cross-sectional plane of the growth plate was selected as the first plane. Subsequently, we identified a second cross-sectional plane, such that the remaining trabecular bones were completely included between the 2planes. Using the control group as a baseline reference, we observed a 7.52% reduction in vertical length in the low-Mg mice.

### Calculations of quantitative morphological parameters

The micromorphological parameter results in Table 2 reveal decreases in the total tissue volume (5.30%), bone volume (27.69%), percent bone volume (22.93%), fractal dimension (4.27%), number of trabecular segments (29.03%), trabecular segment density (18.47%), number of connecting nodes (27.85%), connecting node density (17.93%), BMD (17.38%), and BMC (22.33%).The table also reveals an increase in the structural model index (9.65%) and surface-area-to-volume ratio (6.83%), as well as nearly no change in the mean trabecular segment radius and mean trabecular segment length. Our calculations showed that the coefficients of variation for total tissue volume with the basal and low magnesium group were 3.82% and 4.90%, respectively; bone volume 34% and 15%; percent bone volume 31% and 18%.

**Table 2:** Quantitative parameters for the imaging assessment of the bone characteristics of mice with a basal or low-Mg diet.

Parameters	Total tissue volume	Bone volume	Percent bone volume	Fractal dimension	Structural model index	Trabecular segment number	Trabecular segment number density
Unit	mm^3^	mm^3^	%				mm^-3^
Basal diet	3.68±0.06	0.36±0.04	9.75±1.17	2.11±0.02	2.38±0.05	1119±225	5507±560
Low-Mg diet	3.48±0.09*	0.26±0.02*	7.51±0.59*	2.02±0.01*	2.61±0.04*	794±73*	4490±176*
Parameters	Mean trabecular segment radius	Mean trabecular segment length	Connecting node number	Connecting node density	Surface area to volume ratio	BMD	BMC
Unit	mm	mm		mm^-3^	mm^-1^	mg-HA/cm^3^	mg-HA
Basal diet	0.0140±0.0002	0.1355±0.0021	704±127	3530±372	80.39±4.43	40.07±4.55	149.01±19.09
Low-Mg diet	0.0139±0.0003	0.1359±0.0022	508± 39*	2897±140*	85.88±1.73*	33.10±2.80*	115.74±10.49*

Data are given as the mean ± SEM (n = 8).

All data were subjected to a *t* test; *P* < 0.05 was considered the level of statistical significance.

* *P* < 0.05 (compared with the basal diet group).

### Distributions of the trabecular segment length and radius

**Fig 3:**
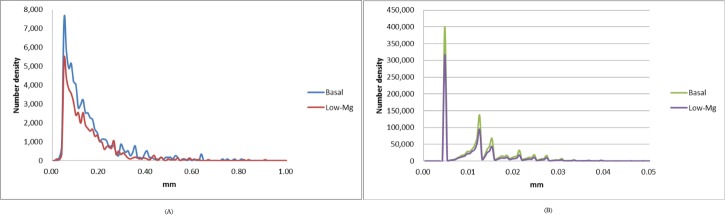
**Distributions of trabecular segment (A) length and (B) radius in mice with a basal or low-Mg diet.** The curves have not been normalized, and the area under the distribution curve represents the total number of trabecular segments.

We examined the distributions of the trabecular segment length (Fig 3A) and radius (Fig 3B). A distribution is a histogram of a probability density function for a specific measurement. The mean trabecular segment lengths and radii of the trabecular bones were obtained from the distributions. The mean length (radius) of the trabecular segments was 0.1355 ± 0.0021 mm (0.0140 ± 0.0003 mm) for the control group and 0.1359 ± 0.0022 mm (0.0139 ± 0.0003 mm) for the low-Mg group. Our imaging assessment indicated that the differences in the magnitudes of the mean length and radius of the trabecular segments at the distal metaphysis were nonsignificant between the 2 groups. In addition, we identified a global maximum of 0.0505 mm for the trabecular segment length and a series of local maximums for the trabecular segment lengths by examining the histograms.

### Biochemical analysis

A biochemical analysis was also conducted in this study. Four-week-old C57BL/6J male mice were supplied with a basal or low-Mg diet. After 8 weeks, the mice were placed into metabolic cages for 24h to collect urine. Subsequently, the mice were sacrificed, and blood was collected for a biochemical analysis. Table 3 shows the concentrations of Mg, Ca, and P in the plasma. The average concentrations of Mg in the plasma were 2.27±0.03 and 1.29±0.04 mg/dL in the control and low-Mg mice, respectively. The Mg concentration in the plasma was significantly 43%lower in the low-Mg group than in the control group. The Ca concentration in the plasma was 7.73±0.57 and 4.66±0.11 mg/dL in the control and low-Mg groups, respectively, representing a 39% decrease in the low-Mg group. No significant difference was observed in the concentration of P in the plasma between the 2 groups. However, the biochemical analysis results showed that the low-Mg diet resulted in a reduction in both Mg and Ca levels in the plasma.

**Table 3:** Plasma concentrations of Mg, Ca, and P in mice with a basal or low-Mg diet.

Plasma levels	Basal diet	Low-Mg diet
Mg (mg/dl)	2.27±0.03	1.29±0.04*
Ca (mg/dl)	7.73±0.57	4.66±0.11*
P (mg/dl)	5.89±0.27	5.36±0.29

Data are given as the mean ± SEM (n = 8).

All data were subjected to a *t* test; *P* < 0.05 was considered the level of statistical significance.

* *P* < 0.05 (compared with the basal diet group).

### Mg, Ca, and P concentration levels in urine

Table 4 shows the Mg, Ca, and P levels in the urine. The table shows that the concentration of Mg in the urine was 0.49±0.05 and 0.02±0.00 mg/dL in the control and low-Mg groups, respectively. The level of Mg in the urine in the low-Mg group was 96% lower than that in the control group. The concentration of Ca was 0.14±0.02 and 0.04±0.01 mg/dL in the control and low-Mg groups, respectively. The level of Ca in the urine in the low-Mg group was 71% lower than that in the control group. Moreover, both Mg and Ca in the urine samples were significantly lower in the low-Mg group than in the control group. The mean concentration of P in the urine was 3.92±0.16 and 4.11±0.33 mg/dL in the control and low-Mg groups, respectively. The concentration of P in the urine was higher in the low-Mg group than in the control group. Thus, the low-Mg diet reduced both Mg and Ca levels in the plasma and urine.

**Table 4:** Urinary excretion of Mg, Ca, and P in mice with a basal or low-Mg diet.

Urinary excretion	Basal diet	Low magnesium diet
Mg (mg/day)	0.49±0.05	0.02±0.00*
Ca (mg/day)	0.14±0.02	0.04±0.01*
P (mg/day)	3.92±0.16	4.11±0.16*

Data are given as the mean ± SEM (*n* = 8).

All data were subjected to a *t* test; *P*< 0.05 was considered the level of statistical significance.

* *P*< 0.05 (compared with the basal diet).

## Discussion

Our imaging assessment revealed a lower population of femoral trabecular bones at the distal metaphysis in the low-Mg mice (Fig 2). The microstructural parameter of total tissue volume represents the overall volume of the ROI, and the image analysis indicated a 5.30% decrease in the low-Mg mice. Bone volume represents the volume of the trabecular bones in the ROI, and our calculations revealed a 27.69% decrease in the low-Mg mice. The decrease in trabecular bone volume may indicate that the functional activity between osteoblasts and osteoclasts was modified in the low-Mg mice. Percent bone volume represents the percentage portion of bone volume in total tissue volume, and our calculations indicated a 22.93% decrease in the low-Mg mice. The fractal dimension represents the surface complexity in a noninteger dimension, and our calculations revealed a 4.27% decrease in the low-Mg mice. The structural model index represents the relative prevalence of rods and plates in a given 3-dimensional structure. Our calculations revealed an increase of 9.65% in the low-Mg mice, indicating a structural transition in micromorphology from a geometric plate-like architecture to a rod-like architecture in the low-Mg mice.

In this study, we considered the structure of the trabecular bones to be interconnected by trabecular segments. A trabecular segment was then modeled as a cylindrical rod with a specific radius and length. A connecting node was defined as a branch node interconnected by various trabecular segments. The number of connecting nodes was considered to represent the population of trabecular bones. Our calculations indicated a 29.03% decrease in the number of trabecular segments, a 18.47% decrease in the trabecular segment density, a 27.85% decrease in the number of connecting nodes, and a 17.93% decrease in the connecting node density in the low-Mg mice.

The surface-area-to-volume ratio represents the amount of surface area per unit of volume. For objects of a similar shape, the value of the surface-area-to-volume ratio is inversely proportional to the size [26,27]. The ratio between the surface area and volume of trabecular bones is a critical measure in biology, particularly for cells with specific shapes, and can be considered to correlate the functional dynamics of osteoblasts and osteoclasts [26–28]. Our calculations revealed an increased ratio of surface area to volume in the low-Mg mice, suggesting that the global size of the trabecular bones was smaller in the low-Mg mice.

Two essential features of the geometric structure of trabecular segments are the magnitudes of the length and radius, specifically because the trabecular segment is considered a cylindrical rod. Fig 3 shows similar distributions, and our quantitative calculations revealed nonsignificant differences in the magnitudes of the average length and radius of the femoral trabecular bones at the distal metaphysis between the 2 groups. Therefore, our findings regarding the local assessment of the femoral trabecular bones indicate that the geometric shape of the femoral trabecular bones was similar in both groups. However, fewer femoral trabecular bones were observed in the low-Mg mice. Consequently, the total volume and population of trabecular bones were decreased in the low-Mg mice.

The trabecular segment length and segment radius were 0.0–0.9 mm and 0.0–0.04 mm, respectively. A global maximum was identified at 0.05051 mm for the trabecular segment length. A series of local maximums of trabecular segment radii was identified at various lengths of 0.004675, 0.012375, 0.015125, 0.018425, 0.021175, 0.024475, 0.027225, 0.030525, and 0.033275 mm. This novel finding is not discussed in the literature. Fundamental kinetics of biology and physics for femur trabecular bones may be established from the distributions of the trabecular segment length and radius. Therefore, further investigation is required to understand the relationship between our findings regarding the local maximums in the length and radius of trabecular bones and their corresponding biological consequences.

Bones are the major reservoir of minerals—Ca and Mg in particular. Adequate BMD and BMC are considered essential to bone quality and strength for maintaining biomechanical function [29]. In the present study, we observed a 17.38% decrease in BMD and a 22.33% decrease in BMC in the low-Mg mice. In addition to Mg, Ca is a critical mineral for the development of healthy bones. Our biochemical analysis of the plasma (Table 3) and urine (Table 4) revealed 39% and 71% decreases in Ca in the plasma and urine, respectively, in the low-Mg mice. Thus, our analysis suggests that the amount of Ca which remains in low-Mg mice may be higher than normal mice.

The 16-bit grayscale images of micro-CT are typically presented in Hounsfield units or CT numbers [30]. In this study, Hounsfield units were calibrated using water as a reference. Hounsfield units are directly associated with a linear attenuation coefficient and correlate linearly with both electron and physical density. Electron and physical density are essential quantities for contrast detection in micro-CT imaging. In clinical applications, Hounsfield units are represented by integers ranging between -1000 and 3000. In radiation physics, Hounsfield units correspond to the interaction between X-ray photons and matter, including the photoelectric effect and Compton scattering [12,30]. In the present study, we used an energy setting of 50 kVp to improve the image contrast and segmentation accuracy, a spatial resolution of 9 μm to reduce image artifacts associated with the partial volume effect, and image processing to correct for beam hardening in order to reduce cupping artifacts.

Many factors will potentially affect the image quality of micro CT; hence, the accuracy of our quantitative analysis was limited due to these factors, such as image artifacts, reconstruction algorithms, imaging settings, and image processing methods. Moreover, some artifacts of micro CT images include beam hardening, ring, cupping, and partial volume artifacts. Here in our work, we applied a post-correction method in the CTAn program to reduce the impact of beam harening and ring artifacts on the image quality.

## Conclusions

The quality of trabecular bones can be determined according to their BMD and architectural characteristics. Our technical assessment performed using micro-CT imaging quantitatively revealed a lower population of trabecular bones at the distalmetaphysis in mice that were supplied a low-Mg diet.Compared with mice that were supplied a basal diet, the low-Mg mice exhibited a decrease in the total tissue volume, trabecular bone volume, percent bone volume, fractal dimension, number of trabecular segments, trabecular segment density, number of connecting nodes, connecting node density, and BMC,as well as an increase in the structural model index and surface-area-to-volume ratio in the low-Mg mice.We also examined the distributions of the trabecular segment length and radius, which havenot been discussed in previous research. A global maximum of the trabecular segment length and a series of local maximums of the trabecular segment radius were identified in the present study. In our experiments, equal amounts of Ca were supplied to both groups. However, our biochemical analysis revealed lower concentrations of Ca in the plasma and urine of the low-Mg mice. Consequently, additional Ca may have been retained in the low-Mg mice.Our findings indicate that retention of Ca may be regulated by adjusting the amount of dietary Mg. Further study is required to clarify the physical and biological interactive mechanisms of Ca–Mg and the corresponding effect on bone growth.
